# Common elements-based nurturing care intervention to improve mother–infant responsive interaction and maternal psychological wellbeing in rural Pakistan: findings of a single-blinded individual randomised controlled trial

**DOI:** 10.1136/bmjopen-2026-119908

**Published:** 2026-10-08

**Authors:** Syed Usman Hamdani, Zill-e Huma, Ayella Gillani, Huda Jamil, Syeda Wajeeha Zafar, Lubna Ejaz Kahloon, Fakhira Shafique, Hashim Javed, Duolao Wang, Atif Rahman

**Affiliations:** 1 Global Institute of Human Development, Shifa Tameer-e-Millat University, Islamabad, Islamabad Capital Territory, Pakistan; 2 Global Institute of Human Development, Shifa Tameer-e-Millat University, Islamabad, Punjab, Pakistan; 3 Liverpool School of Tropical Medicine, Liverpool, Liverpool, UK; 4 Institute of Population Health, University of Liverpool, Liverpool, UK

**Keywords:** MENTAL HEALTH, Caregivers, Social Cognition, Community child health

## Abstract

**Objectives:**

To evaluate the effectiveness of a common elements–based Nurturing Care for Early Child Development Intervention delivered by non-specialists in improving mother–infant responsive interaction at 12 months postpartum among pregnant women experiencing psychological distress in rural Pakistan.

**Design:**

Two-arm, single-blinded, individually randomised controlled trial.

**Setting:**

Community-based primary care settings in a rural sub-district of Rawalpindi, Pakistan.

**Participants:**

Pregnant women in their third trimester aged 18–40 years who screened positive for psychological distress (Self-Reporting Questionnaire score ≥9). Of 1962 women assessed for eligibility, 250 were enrolled and randomised to the intervention (n=125) or treatment-as-usual (TAU) arm (n=125).

**Interventions:**

Participants in the intervention arm received 15 monthly individual sessions of a Nurturing Care for Early Child Development intervention delivered by trained non-specialist facilitators from the third trimester of pregnancy until 12 months postpartum. The intervention included early stimulation, learning through play, responsive feeding, guided discovery using pictures, behavioural activation and problem-solving. Participants in the control arm received routine maternal and child health services (treatment as usual).

**Primary and secondary outcome measures:**

The primary outcome was caregiver–infant responsive interaction measured using the Observation of Mother–Child Interaction (OMCI) at 12 months postpartum. Secondary outcomes included maternal psychological well-being, quality of life, functioning and perceived social support, as well as infant growth including birth weight, nutrition, cognitive development and socio-emotional development. Data were collected at baseline (third trimester), 6 months and 12 months postpartum. Endpoint assessments were delayed by 2 months due to COVID-19 lockdown restrictions.

**Results:**

Of the 250 participants randomised, 206 completed the 12-month follow-up assessment. At the endpoint, mother–infant dyads in the intervention arm demonstrated significantly higher OMCI total scores compared with TAU (mean (SD) 42.05 (8.65) vs 29.74 (8.19); adjusted mean difference 12.31, 95% CI 9.99 to 14.62; p<0.0001). As a secondary outcome, infants in the intervention arm had higher birth weight (mean difference 0.78 kg, 95% CI 0.30 to 1.25; p<0.001). Improvements were also observed in infant cognitive and socio-emotional development and maternal psychological well-being, functioning and quality of life.

**Conclusions:**

A common element–based nurturing care intervention delivered by trained non-specialists improved mother–infant responsive interaction among infants of mothers experiencing psychological distress.

**Trial registration number:**

NCT04252807.

**STRENGTHS AND LIMITATIONS OF THIS STUDY**

- This study used a single-blinded, individual randomised controlled trial design with prospective registration and a pre-published protocol, strengthening methodological rigour and transparency.
- The intervention integrated common evidence-based elements from multiple early childhood development and maternal mental health programmes, targeting improving responsive caregiving practices.
- Task-sharing with trained, supervised, and competent non-specialist facilitators enabled delivery of the intervention within the MNCH system in community settings.
- Multiple validated instruments were used to measure maternal and infant outcomes, including direct observation of mother–infant interaction and standardised developmental assessments, enhancing measurement reliability.
- Trial procedures were adapted during the COVID-19 pandemic, including delays in endpoint assessments and partial telephone-based intervention delivery, demonstrating the feasibility of telephonic intervention delivery.

## Background

 About one in six children under the age of five in low- and middle-income countries (LMICs) are exposed to a higher risk of suboptimal development.^1^ A systematic review and meta-analysis of studies in LMICs estimated that about 18.83% of children under five have developmental delay.^2^ Risk factors, such as maternal distress, inadequate age-appropriate stimulation and malnutrition, pose a significant threat to the optimal development of young infants, leading to poorer cognitive functioning and socio-emotional development outcomes.^3^ It has been consistently reported that providing responsive caregiving during the first 3 years of life is associated with improved physical, cognitive and socio-emotional outcomes in children, regardless of the income level of their country.^4^

In Pakistan, more than 20 per cent of the population lives on less than US$1.25 (purchasing power parities, PPP) per day. Pakistan is among the countries with the highest under-five mortality rates (ie, 65.2 per 1000 live births). There is a noticeable absence of comprehensive data on early childhood education programmes for caregivers and responsive caregiving in Pakistan;^5^ however, early child development is gradually gaining prominence on the Pakistani government’s agenda. A pivotal development is the formulation of the National Health and Population Policy (NHPP) 2025–34^6^, which highlights the importance of enhancing the health and well-being of vulnerable segments of the population, particularly women and children, by integrating health and population strategies into national planning and service delivery. The NHPP aims to strengthen healthcare governance, expand access to essential health and family planning services and align Pakistan’s health system with global health standards to accelerate progress toward the Sustainable Development Goals (SDGs) and population outcomes. To achieve this vision, substantial efforts have been made in child survival programmes, resulting in a reduction in child mortality rates over the past decade in Pakistan.^7 8^ However, the socio-political landscape in Pakistan, while more intricate, mirrors the situation in many low-income countries. Moreover, healthcare systems in Pakistan face considerable challenges, with limited resources significantly impeding publicly funded primary healthcare initiatives. Consequently, at-risk mothers and infants in Pakistan constitute a highly disadvantaged group with unmet healthcare needs.

Recognising the paramount importance of the initial 3 years in fostering positive early child development, particularly in low resource settings, the WHO, UNICEF and the World Bank collaboratively launched the ‘Nurturing Care Framework’ (NCF).^9^ This framework serves as a guiding blueprint, outlining strategic actions for governments and policymakers to ensure the provision of nurturing care for young children. Within this framework, responsive caregiving is positioned as a foundational mechanism through which early experiences shape developmental trajectories. Responsive caregiving strengthens caregivers’ ability to recognise and interpret their child’s cues and respond promptly, sensitively, and in developmentally appropriate ways through “serve-and-return” interactions.^10^ By strengthening caregivers’ skills and confidence, the NCF seeks to translate knowledge into consistent, emotionally supportive daily interactions. This, in turn, facilitates the creation of an enabling environment and early learning opportunities for children through responsive and emotionally supportive interactions.^11^

Despite growing recognition of both effective intervention components and the contextual determinants influencing caregiving practices, implementation remains challenging. These interventions are often delivered through distinct and overlapping packages within fragmented systems and face numerous implementation barriers, including shortages of trained human resources and inadequate mechanisms for training and supervision.^12^ Such structural constraints limit the potential for sustained impact and hinder efforts to institutionalise nurturing care within existing service platforms. These challenges are particularly pronounced in resource-constrained settings, where evidence remains unevenly distributed across regions and populations, with most studies conducted in high-income contexts and limited synthesis from low-resource settings.^13^ Together, these gaps highlight the need for contextually grounded, workforce-supported models of nurturing care that are rigorously evaluated in low-resource environments.

To address these barriers, using the principles of integrated innovation,^14^ we developed a non-specialist-delivered, Nurturing Care for Early Child Development (NC-ECD) intervention. This intervention combines evidence-based strategies with culturally tailored approaches to promote early child development, while leveraging non-specialists to overcome implementation barriers in low-resource settings in Pakistan.

The aim of the present study was to evaluate the effectiveness of non-specialists delivering NC-ECD intervention compared with usual care in improving mother-infant responsive interaction as well as improvements in maternal psychological well-being, infant growth, nutrition and development at 12 months postpartum in low-resource rural community settings in Pakistan.

## Methods

### Study design

A two-arm, single-blinded, individual randomised controlled trial was conducted in community settings in the rural sub-district of Gujar Khan, Rawalpindi district, Pakistan. The study was completed over a period of 18 months. Ethics approval of the study was obtained from the Institutional Review Board of the Human Development Research Foundation (HDRF) (ethics approval certificate number IRB/005/2019). The full trial protocol is available online (*see online supplemental material A, trial protocol*).^15^ The trial was registered prospectively under NCT04252807 at clinicaltrials.gov. Similar to challenges faced in other global health trials,^16^ the COVID-19 pandemic resulted in a 2-month delay in endpoint assessments in the current trial, required adjustments to data collection procedures (including a partial shift from in-person to telephone-based engagement) and contributed to delayed analysis and reporting of the trial.

### Study settings and participants

The present study was conducted in a sub-district of Gujar Khan in the Rawalpindi district, Pakistan (an approximate population of 1 million). The sub-district is typical of rural Pakistan, with poor demographic and health profile indicators (high fertility rate—four births per woman and low female literacy rate, ie, 45%),^17^ and recent evidence suggests that a substantial proportion of pregnant women in rural Pakistan experience prenatal depression (with national antenatal depression prevalence estimates around 37% in community-based studies).^18^ An estimated infant mortality rate in Rawalpindi is 55 per thousand live births, the under-five mortality rate is 82 per thousand live births, the percentage of underweight children below 5 years of age is 25% and rates of moderate to severe stunting are 16% and 5%, respectively.^19^

Primary healthcare is delivered through a network of Basic Health Units (BHUs), each of which provides care to about 15 000–20 000 people. Each BHU is staffed by a doctor, midwife, vaccinator and 15–20 village-based community health workers (CHWs), also called lady health workers (LHWs). These women have completed secondary school and are trained to provide preventive maternal and child healthcare and education in the community. Each LHW is responsible for about 100 households in her village.

The participants were pregnant women in their third trimester, aged 18–40 years, living in the community setting of the study sub-district for the duration of the study. The participants were recruited from the catchment area of LHWs, where lists of all potential participants were compiled from their official registers. All potentially eligible participants were screened for psychological distress on the Self-Reporting Questionnaire (SRQ, cut-off score ≥9) by the research staff until the desired enrolment number was reached. Women who required immediate or on-going medical or psychiatric care, had serious previous or current obstetric morbidity, including eclampsia and antepartum haemorrhage, and/or medical disorders that required inpatient management (eg, diabetes, hypertension, thromboembolism, cardiac disease), were excluded. Written informed consent or independently witnessed consent (where applicable) from all study participants was obtained before participation in the research study.

### Randomisation and blinding

The participants were randomised into the intervention or control arm on a 1:1 basis using computerised software by an independent researcher. The random sequence was kept in sequentially numbered, opaque, sealed envelopes at the off-site centre to ensure its concealment. Only the assessment team (conducting baseline and follow-up assessment) was blind to the treatment allocation of trial participants. To maintain the blinding, all trial participants were briefed prior to the assessment about the significance of blinding and the importance of not disclosing their allocation status to the assessment team members. The fidelity of the masking was assessed by asking the assessors to guess the allocation status of each trial participant after the post-intervention assessment.

### Interventions

#### Common elements-based ‘Nurturing Care for Early Child Development’ intervention

The intervention called ‘Nurturing Care for Early Child Development’ combines common elements from evidence-based interventions for maternal psychosocial distress (eg, WHO Thinking Healthy Programme,^20^ early stimulation and responsive feeding (eg, UNICEF Care for Child Development Package)^21^ and childcare (eg, WHO Caregivers Skills Training programme)^22^ for optimal development of at-risk mothers and children. By combining overlapping theoretical approaches into a unified model, the intervention aims to generate synergistic benefits across multiple developmental domains, including early stimulation, responsive caregiving, nutrition and maternal well-being.

These approaches recognise the importance of the early critical years and the role of the primary caregiver in providing nutrition and an optimal environment for interaction and stimulation. The intervention package targets improving (a) maternal psychological distress by promoting problem solving, developing empathic relationships, improving family support, using guided discovery through pictures and behavioural activation; (b) child development through psycho-education and responsive breastfeeding; and (c) mother-infant interaction through early stimulation and learning through play. These components are aligned with common practice elements identified across effective LMIC early childhood development programmes, particularly responsive caregiving, play and communication strategies and structured caregiver psychoeducation.^23^ The intervention focuses on providing caregivers (usually mothers) with psychosocial support, education and skills training related to early child development through a well-monitored and supervised delivery system. Nutrition and child health components included guidance on the introduction of solid foods, the concept of ‘super foods', food and hygiene practices, continuation of breastfeeding with weaning at 6 months and the importance of vaccination. The NC-ECD intervention was co-designed in collaboration with members of the local community, including mothers with lived experience of post-natal depression, fathers, extended family, Community Health Workers (CHWs) and local volunteers, before implementation.^15^ It is designed to be delivered by non-specialist facilitators in 15 monthly individual sessions in community settings, beginning in the third trimester of pregnancy and ending 12 months after birth.

### Training of non-specialist facilitators

The delivery agents of the intervention were female non-specialist facilitators who were graduates in psychology or social sciences with little or no prior experience in delivering psychosocial interventions to mothers with perinatal distress and their newborn infants. A cascade model of training and supervision was followed to deliver the intervention. The training was conducted in a classroom setting. The programme trainers trained 15 non-specialist facilitators. The training consisted of 10 days of classroom teaching and training on the basic theoretical concepts of the ‘Nurturing Care’ intervention, its strategies, group discussions and role-plays, followed by practice cases under the supervision of the programme trainer. The participants of this practice group were two pregnant women without distress and their infants, selected from the same community after seeking informed consent for participation in the training. The programme trainer performed a live competency rating during practice cases using an adapted version of the ENhancing Assessment of Common Therapeutic (ENACT) tool for NC ECD intervention.^24^ Only competent non-specialist facilitators (mean score above 3 (±0.5) on all domains of the adapted ENACT 17-item tool) were allowed to deliver the intervention.

#### Fidelity assessment

Each non-specialist facilitator was given a caseload of 8–10 pregnant women to deliver intervention. The fidelity of the intervention was evaluated using an adapted ENACT tool developed for the programme. The programme trainer directly observed and rated 20% of randomly selected intervention sessions (n=360, 24 sessions per non-specialist facilitator) at different stages of the intervention delivery (during the implementation of Modules 1, 2 and 3 separately) to assess the fidelity of intervention delivery by non-specialist facilitators.

### Supervision

Programme trainers were experts in mental health and infant development and provided weekly supervision (2 hours) to non-specialist facilitators to ensure intervention fidelity. Supervision involved discussion of difficulties encountered in delivering interventions as well as the self-care of the non-specialist facilitators. Over the period of 15 months, 60 supervision meetings were conducted (four sessions per month).

### Treatment as usual (TAU)

Participants and their family members in both study arms received maternal and childcare services from LHWs in their respective areas as part of the usual care. This included receiving psychoeducation about family planning and maternal and child health; receiving free contraception and iron-folic acid supplements; receiving support and monitoring during pregnancy, childbirth and new-born baby’s progress on growth parameters (weight and height); and referral services if required. A complete record of services received by trial participants in both study arms was obtained using the Client Services Receipt Inventory (CSRI).

### Outcomes

The primary outcome was caregiver–child interaction (CCI), assessed by live rating of mother–child interaction using the Observation of Mother and Child Interaction (OMCI) tool at 12-month postpartum. Based on a theoretical framework by Landry, Smith and Swank,^25^ this tool has been designed to measure the frequency of four domains of maternal responsive behaviours (contingent responding, emotional-affective support, support for infant foci of attention and language inputs) through a structured interaction activity. This tool comprises 19 items related to maternal and child behaviours. Scores are based on the frequency of observed maternal and child behaviours, with higher scores indicating more responsive interaction. Maternal behaviours include components of maternal affect, touch, verbalisation, sensitivity and contingent responses, scaffolding, language stimulation and focus, whereas child outcomes include child affect, child focus, the child’s communication efforts and mutual enjoyment.

Interventions focusing on responsive stimulation have been shown to improve child outcomes^26–31^ indicating that responsive caregiving is a modifiable domain of focus. Therefore, the OMCI may be an ideal tool to guide interventions and studies geared towards optimising child development through responsive caregiving. The tool has been extensively tested with a similar population in Pakistan and has been shown to be sensitive to detecting changes in mother-child interaction as a result of responsive caregiver intervention at 12- and 24-month postpartum.^32^ In the present study, the research team observed a live 5 min mother–infant interaction while the mother and infant played together using a picture book.

The infant secondary outcomes included the following:

- *Bayley’s Scales of Infant Development (BSID III*) to measure cognitive, language, motor, socio-emotional development and adaptive behaviour at 12 months postpartum. The tool has been used previously in similar South Asian settings.^31^
- *Ages and Stages Questionnaire, third Edition (ASQ3*)^31^ to measure developmental delays in communication, gross motor, fine motor, problem solving and personal social domains at 12 months’ postpartum. It provides a cut-off score in five domains of development that indicates a possible need for further evaluation. It highlights results that fall into a “monitoring zone” to make it easier to keep track of children at risk. The tool has been validated for use in a South Asian community setting.
- Quality and quantity of stimulation and support in the child’s home were measured using the *Home Inventory* at 12 months’ postpartum.^30^ The tool has been validated for use in Pakistan previously.^31^
- Infant weight (in kg) at birth was obtained from the birth registration card retrospectively at endpoint. The infant anthropometric measurement date at 12-month postpartum was not obtained due to restrictions caused by the COVID-19 pandemic.^33^
- The data on *early breastfeeding practices* at 6-month postpartum and on *diarrhoeal episodes and infant immunisation* were also obtained at 6 and 12-month postpartum.

For maternal secondary outcomes, the *Self-Reporting Questionnaire (SRQ*) was used to assess maternal distress at baseline, 6-month and 12 month postpartum. SRQ is a 20-item self-report measure to detect non-specific psychological distress, developed by the WHO.^34^ The SRQ items are scored 0 or 1. A score of 1 indicates the presence of symptoms of psychological distress during the past month, and a score of 0 indicates the absence of symptoms. The higher score indicates the presence of higher psychological distress. A score of more than 9 indicates an increased risk of postpartum depression or maternal distress.

Mothers’ health-related quality of life was measured by the WHO Quality of Life (WHOQoL)-Brief Version ^35^ at baseline and 12-month postpartum. WHOQoL assesses the participants’ position in life in the context of the culture and value systems in which they live and in relation to their goals, expectations, standards and concerns. WHOQoL comprises four domains, namely physical health, psychological health, social relationships and environment. Items are rated on a 5-point Likert scale, where 1 represents ‘disagree’ or ‘not at all’ and 5 represents ‘completely agree’ or ‘extremely’.

The level of social support was measured using the Multi-Dimensional Scale of Perceived Social Support (MSPSS)^36^ at baseline and 12-month postpartum. It includes 12 items that cover three dimensions: support from family, friends and a significant other. Each item is rated on a 7-point Likert scale (1=very strongly disagree; 7=very strongly agree). A total score is calculated by summing the responses to all items (ranging from 12 to 84), with higher scores indicating higher levels of perceived social support.^37^

The caregiver’s level of functioning was measured using the WHO Disability Assessment Schedule (WHODAS)^38 39^ at baseline and 12 months’ postpartum. WHODAS assesses participants’ health-related difficulties in six domains of life over the previous 30 days (including understanding and communicating; moving and getting around; attending to one’s hygiene; dressing, eating and staying alone; interacting with others; domestic responsibilities; leisure, work and school; and participating in community activities and society). *Maternal financial empowerment* was measured at baseline and 12 months postpartum using a questionnaire consisting of (a) two items previously used in the same population that assess financial empowerment (ie, *whether a woman is given a lump sum of money for day-to-day expenses and whether she can make independent decisions about its use*). Women who answer *‘yes*’ to both items on financial empowerment are classified as ‘empowered’.

The *Client Service Receipt Inventory* was administered to record health services accessed by trial participants at 12-month postpartum. The tool has been adapted previously for use in Pakistan.^39^

The implementation of the trial overlapped with the COVID-19 pandemic, which necessitated procedural modifications, including temporary pauses in field activities, delays in endpoint assessments, and a partial shift from in-person to telephone-based intervention delivery. These adjustments were introduced to maintain participant safety and ensure continuity of trial procedures within public health restrictions. Adverse events were assessed at each outcome assessment time point.

### Statistical analysis

The study was powered to make a single comparison in the primary outcome at 12 months only. The effect size estimates for this study were based on the findings of a similar study conducted in Pakistan, where OMCI (the primary outcome in this study) was used as one of the outcomes to evaluate the effect of responsive stimulation intervention on caregiver–child interaction at 12-month postpartum.^40^ Because the effect sizes for OMCI in study^41^ ranged from 0.6 to 0.9 at 12-month postpartum, a conservative effect size of 0.4 (two-sided hypothesis) at 12-month post-partum was considered meaningful in the current study to detect treatment effects. The effect size was calculated using the two-sided t-test. Assuming an effect size of 0.4 with 80% power, 0.05 significance, a two-sided hypothesis test, and accounting for 20% attrition, 250 mother-infant dyads, equally randomised to the intervention and control arms (125 in each arm), were included in the study. The data was analysed using an intent-to-treat approach.

For the analysis of the primary outcome (mother-infant responsive interaction across two arms at 12 months post-partum), a linear regression model was employed with treatment as the sole predictor. In addition, adjusted linear regression model analysis was performed with the pre-specified covariates (gender of the infant, mother’s education and severity of mothers’ psychosocial distress measured using the SRQ) measured at baseline and added into the above linear model. The crude and adjusted mean differences in the primary outcome, together with their 95% CIs at 12 months, were derived from the linear models. In addition, subgroup analysis of the primary endpoint was performed on the above pre-specified covariates.

The analysis of secondary continuous outcomes with a single follow-up measurement was done in a similar fashion to the primary endpoint analysis. Analysis of secondary continuous outcomes with repeated follow-up measurement was performed using a linear mixed model with treatment visit, interaction between treatment and visit as fixed effects, the baseline value of the outcome as a covariate, if available, and subject as random effects. Missing primary and secondary outcomes were not imputed in the primary analysis, whereas missing secondary outcomes with repeated measurements were imputed using the ‘last observation carried forward’ strategy in sensitivity analysis. Missing baseline covariates were imputed using simple imputation methods in the covariate-adjusted analysis based on the covariate distributions in the sample. SAS 9.4 was employed for the statistical analyses.

### Procedure

The study participants were screened between June 2019 and August 2019 from the catchment area of LHWs involved in the study with the first participant providing informed consent and being enrolled on 7 February 2020, which marks the start of participant data collection. Participants meeting the eligibility criteria were invited to be enrolled in the study. Written informed consent for participation in the study was obtained before data collection by the research team. Following enrollment, participants were randomly assigned to one of two arms: intervention or control (125 in each). Participants in the intervention arm received a total of 15 individual monthly sessions of the intervention by a non-specialist facilitator, in addition to TAU from LHWs. The participants in the control arm just received TAU from the LHWs. Post-intervention data was collected at 6 and 12 months postpartum. Participants who developed severe psychiatric problems (eg, psychosis, imminent suicidality) at follow-up were referred to a tertiary mental healthcare facility for specialist care (n=2). As the study was conducted amid COVID-19, all in-person post-intervention assessments were conducted following appropriate precautionary measures.

## Results

The findings of the study are reported following the Consolidated Standards of Reporting Trials guidelines for a randomised controlled trial^42^ (figure 1, Study Flow). About 1962 potentially eligible pregnant women in their third trimester were assessed for eligibility. Out of 1962, 250 women meeting the eligibility criteria were included in the study and were randomly assigned to the intervention and control arms. The follow-up rate at the 12-month postpartum was 84% (105/125) in the intervention arm and 81% (101/125) in the control arm. There were no statistically significant differences between the two arms in demographic characteristics. The mean age of the mother at baseline was 27.03 (±4.90) years, and the mean age of the infant at endpoint was 14.54 (±1.25) months (table 1). About 44% of mothers had completed eighth grade education, and 77% (160/250) were living in a joint family system. Among the 250 infants, 54% were boys.

**Figure 1:**
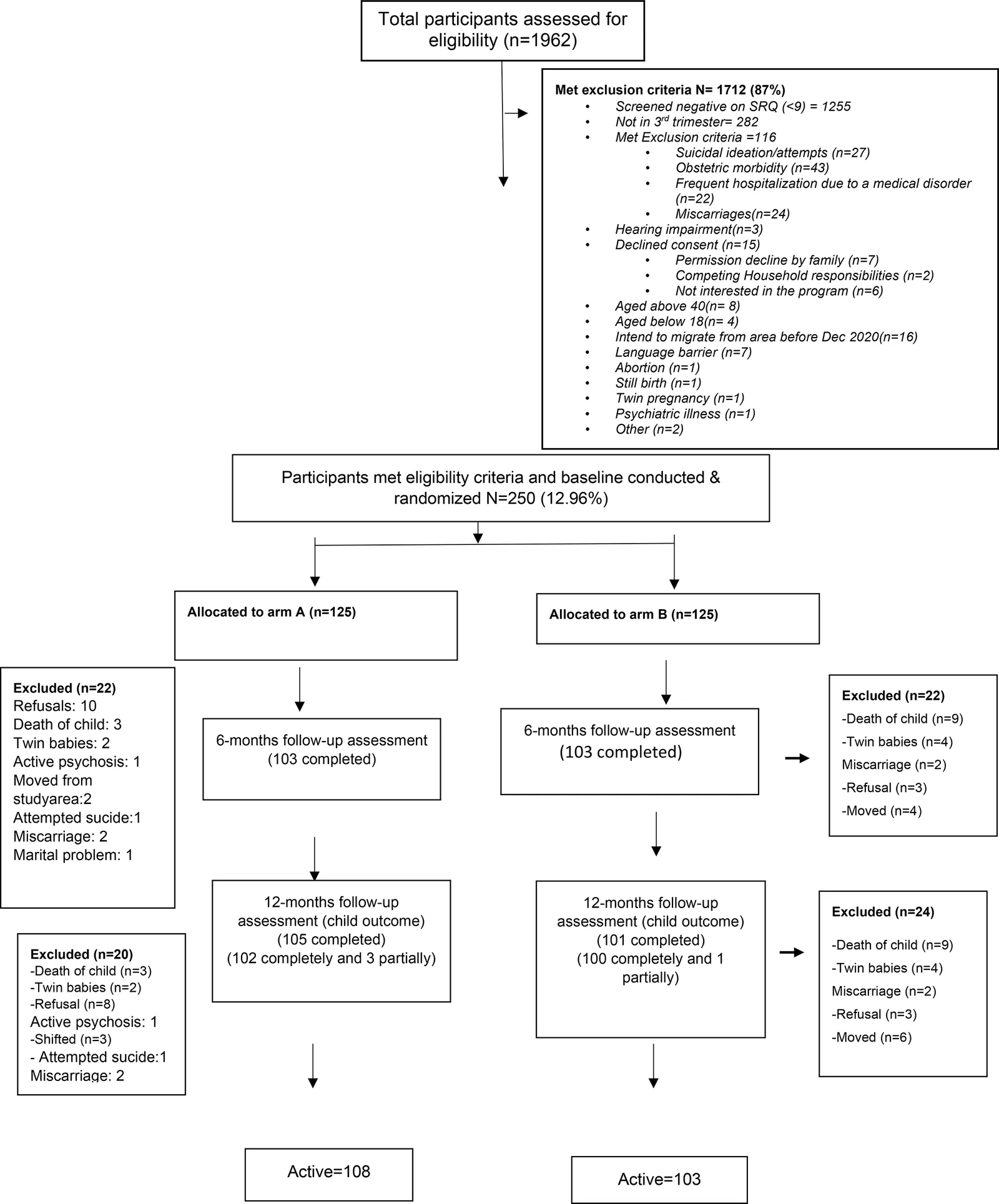
Study flow. SRQ, Self-reporting Questionnaire.

**Table 1:** Demographic characteristics of the study participants and their children (n, %) (n=250)

Variable	Intervention (n=125)	Treatment as usual (n=125)	Total (n=250)
Participant age in years (mean±SD)	27.03±4.90	26.32±4.35	26.68±4.64
Child age in months (mean±SD)	14.54±1.25	14.26±0.95	14.40±1.12
Child gender			
Male	57 (54.3)	49 (48.5)	106 (51.5)
Female	48 (45.7)	52 (51.5)	100 (48.5)
Marriage duration in months (mean±SD)	65.66±52.72	59.99±54.38	62.88±53.49
Mother education			
Uneducated	13 (12.4)	12 (11.9)	25 (12.1)
Completed matriculation (grade 10)	63 (50.4)	61 (48.8)	125
Completed College and University (grade 11–16)	29 (27.6)	27 (26.7)	56 (27.2)
No. of children (mean±SD)	1.44±1.25	1.27±1.27	1.35±1.26
Family structure			
Joint	85 (81.0)	75 (74.3)	160 (77.7)
Nuclear	20 (19.0)	26 (25.7)	46 (22.3)
Employment status of mother			
No	102 (97.1)	99 (98.0)	201 (97.6)
Yes	3 (2.9)	2 (2.0)	5 (2.4)
Father education			
Uneducated	12 (11.4)	8 (7.9)	20 (9.7)
Completed primary (grade 5)	11 (10.5)	11 (10.9)	22 (10.7)
Completed middle (grade 8)	19 (18.1)	27 (26.7)	46 (22.3)
Completed matriculate (grade 10)	44 (41.9)	40 (39.6)	84 (40.8)
Completed College and University (grade 11–16)	18 (17.1)	15 (14.9)	33 (16.0)
Other	1 (1.0)	0 (0)	1 (0.5)
Employment status of father			
No	17 (16.2)	15 (14.9)	32 (15.5)
Yes	88 (83.8)	86 (85.1)	174 (84.5)

Tables 2 and 3 present the findings of primary and secondary outcomes. At 12-month postpartum, mother–infant dyads in the intervention arm, compared with the control arm, had significantly higher responsive interaction scores on OMCI total scores (mean (*SD*), 42.05 (8.65) vs 29.74 (8.19); *Adjusted Mean Difference*, 12.31; *95%* CI, 9.99 to 14.62; p=0.0001) as did individual domain scores on mothers’ behaviour (mean (*SD*), 28.80 (5.43) vs 20.16 (5.41); *AMD*: 8.64; *95%* CI, 7.15 to 10.13; p=0.0001) and child’s behaviour on OMCI (mean (*SD*), 13.25 (3.84) vs 9.58 (3.50); *AMD*: 3.66; *95%* CI 2.65 to 4.67; p=0.0001).

**Table 2:** Summary statistics and results from linear regression model analysis of primary and infants’ secondary outcomes (n=250)

Primary and secondary outcomes	Visit	Intervention (n=125)		Treatment as usual (n=125)		Difference in least squares mean (95% CI)	P value	Effect size
		N	M (SD)	N	M (SD)			
Observation of Mother and Child Interaction (OMCI)								
OMCI Total Score	12 months	105	42.05 (8.65)	101	29.74 (8.19)	12.31 (9.99 to 14.62)	<0.0001	1.46 (1.19 to 1.74)
Mother’s behaviour	12 months	105	28.80 (5.43)	101	20.16 (5.41)	8.64 (7.15 to 10.13)	<0.0001	1.59 (1.32 to 1.87)
Child’s behaviour	12 months	105	13.25 (3.84)	101	9.58 (3.50)	3.66 (2.65 to 4.67)	<0.0001	1.00 (0.72 to 1.27)
Bayley’s Scales of Infant Development (BSID III)—raw scores								
Cognitive	12 months	104	50.12 (4.27)	100	48.46 (5.56)	1.66 (0.29 to 3.02)	0.0178	0.34 (0.06 to 0.61)
Receptive language	12 months	104	14.50 (2.87)	100	13.99 (2.57)	0.51 (−0.24 to 1.26)	0.1831	0.19 (-0.09 to 0.46)
Expressive Language	12 months	104	17.01 (2.96)	100	16.41 (5.23)	0.60 (−0.57 to 1.77)	0.3123	0.15 (-0.14 to 0.43)
Fine motor	12 months	104	29.48 (1.49)	100	29.26 (2.93)	0.22 (-0.42 to 0.86)	0.4964	0.10 (-0.19 to 0.39)
Gross motor	12 months	104	43.84 (3.34)	100	43.92 (3.03)	−0.08 (-0.96 to 0.80)	0.852	−0.03 (-0.30 to 0.25)
Social emotional	12 months	104	75.96 (15.29)	100	67.22 (12.22)	8.74 (4.91 to 12.57)	<0.0001	0.64 (0.36 to 0.91)
Adaptive functioning	12 months	103	31.73 (7.06)	100	28.81 (6.26)	2.92 (1.07 to 4.77)	0.0021	0.44 (0.16 to 0.72)
Adaptive functioning— community use	12 months	103	10.95 (7.04)	100	9.54 (5.72)	1.41 (-0.37 to 3.19)	0.1189	0.22 (-0.06 to 0.50)
Adaptive functioning— Functional Pre-Academics	12 months	103	5.84 (1.78)	100	4.77 (1.84)	1.07 (0.57 to 1.58)	<0.0001	0.59 (0.32 to 0.87)
Adaptive functioning—home living	12 months	103	19.64 (11.16)	100	16.50 (9.77)	3.14 (0.23 to 6.05)	0.0343	0.30 (0.02 to 0.58)
Adaptive functioning—health safety	12 months	103	26.26 (7.85)	100	23.62 (7.43)	2.64 (0.53 to 4.76)	0.0147	0.35 (0.07 to 0.62)
Adaptive functioning—leisure	12 months	103	26.37 (6.34)	100	23.93 (5.64)	2.44 (0.78 to 4.10)	0.0042	0.41 (0.13 to 0.68)
Adaptive functioning—self-care	12 months	103	32.19 (5.99)	100	29.98 (4.99)	2.21 (0.69 to 3.74)	0.0047	0.40 (0.12 to 0.68)
Adaptive functioning—self direction	12 months	103	25.71 (6.55)	100	23.31 (5.56)	2.40 (0.71 to 4.08)	0.0055	0.40 (0.12 to 0.67)
Adaptive functioning—social	12 months	103	30.00 (3.45)	100	28.25 (3.48)	1.75 (0.79 to 2.71)	0.0004	0.51 (0.23 to 0.78)
Adaptive functioning—motor	12 months	103	46.12 (9.34)	100	44.55 (8.15)	1.57 (−0.86 to 4.00)	0.205	0.18 (-0.10 to 0.46)
Home Inventory								
HOME total scores	12 months	105	38.06 (4.61)	100	33.95 (5.65)	4.11 (2.69 to 5.52)	<0.0001	0.80 (0.52 to 1.08)
Home responsivity	12 months	105	9.92 (1.41)	100	8.70 (1.86)	1.22 (0.77 to 1.68)	<0.00001	0.75 (0.47 to 1.03)
Home acceptance	12 months	105	6.77 (1.22)	100	5.46 (1.95)	1.31 (0.87 to 1.76)	<0.0001	0.83 (0.55 to 1.11)
Anthropometric measurements								
Weight of the child (in kg)	At birth	95	2.28 (1.53)	95	1.50 (1.79)	0.78 (0.30 to 1.25)	0.0015	0.47 (0.18 to 0.76)
HAZ	12 months	105	−0.48 (1.76)	101	−0.45 (1.49)	−0.03 (−0.48 to 0.42)	0.8956	−0.02 (-0.29 to 0.26)
WAZ	12 months	105	−0.45 (1.26)	101	−0.59 (1.37)	0.14 (-0.22 to 0.50)	0.4436	0.11 (-0.17 to 0.38)

Effect size was calculated as Cohen’s d, based on the adjusted mean difference divided by the estimated pooled SD.

ASQ-3 comparisons with a total sample size of fewer than 80 participants across the two treatment groups were excluded because the small sample sizes limited the interpretability of the comparisons.

.HAZ, height-for-age z-score; M, mean; SD, standard deviation; TAU, Treatment as usual; WAZ, weight-for-age z-score.

**Table 3:** Summary statistics and results from a linear regression model analysis of maternal secondary outcomes (n=250)

Primary and secondary outcomes	Visit	Intervention (n=125)		Treatment-as-usual (n=125)		Difference in least squares mean (95% CI)	P value	Effect Sizes
		N	M (SD)	N	M (SD)			
SRQ total score*	Baseline	125	11.45 (2.29)	125	11.48 (2.34)			
	12 months	104	2.00 (3.06)	101	3.34 (3.94)	−1.34 (−2.32 to −0.36)	0.0074	−0.38 (−0.66 to −0.10)
MSPSS total scores	Baseline	125	32.79 (6.79)	125	31.64 (7.66)			
	12 months	103	36.16 (5.99)	101	35.08 (6.03)	0.88 (−0.75 to 2.51)	0.29	0.15 (−0.13 to 0.42)
MSPSS Family Subscale	Baseline	125	5.51 (1.50)	125	5.25 (1.60)			
	12 months	103	5.93 (1.39)	101	5.94 (1.17)	−0.02 (−0.38 to 0.33)	0.8964	−0.02 (−0.30 to 0.26)
MSPSS Friends Subscale	Baseline	125	5.15 (1.57)	125	4.87 (1.82)			
	12 months	103	5.75 (1.48)	101	5.40 (1.50)	0.31 (−0.10 to 0.72)	0.1336	0.21 (−0.06 to 0.48)
MSPSS Significant Other Subscale	Baseline	125	5.51 (1.36)	125	5.44 (1.37)			
	12 months	103	6.29 (0.91)	101	6.14 (1.06)	0.14 (−0.13 to 0.41)	0.2949	0.15 (−0.13 to 0.42)
WHODAS complex score	Baseline	125	37.60 (18.89)	125	39.27 (17.68)			
	12 months	103	8.01 (11.96)	101	13.26 (12.74)	−5.24 (−8.66 to −1.82)	0.0029	−0.42 (−0.70 to −0.15)
WHO-QoL Physical Health Transformed Scores	Baseline	125	51.97 (16.51)	125	52.54 (13.95)			
	12 months	103	75.64 (15.22)	101	68.95 (13.04)	6.80 (2.92 to 10.68)	0.0007	0.48 (0.21 to 0.76)
WHO-QoL Psychological Health Transformed Scores	Baseline	125	54.14 (14.32)	125	52.46 (13.82)			
	12 months	123	70.51 (16.52)	101	65.92 (14.72)	4.15 (−0.06 to 8.36)	0.0531	0.27 (−0.00 to 0.53)
WHO-QoL Social Relationship Transformed Scores	Baseline	125	78.39 (17.47)	125	75.45 (18.98)			
	12 months	123	85.90 (17.01)	101	81.01 (16.21)	4.48 (−0.08 to 9.04)	0.0541	0.27 (−0.00 to 0.54)
WHO-QoL Environmental Health Transformed Scores	Baseline	125	61.66 (18.76)	125	60.68 (16.33)			
	12 months	103	70.15 (16.22)	101	64.34 (16.27)	5.49 (1.34 to 9.64)	0.0098	0.34 (0.08 to 0.59)

* A linear mixed model was used to estimate the difference in mean between two groups.

.MSPSS, Multidimensional Scale of Perceived Social Support; SRQ, Self-reporting Questionnaire; WHODAS, WHO Disability Assessment Schedule; WHO-QoL, WHO Quality of Life.

Similarly, at 12-month postpartum, infants in the intervention arm, compared with TAU arm, scored higher on cognitive development (mean (SD), 50.12 (4.27(vs 48.46 (5.56); mean difference: 1.66; 95% CI 0.29 to 3.02; p-value: 0.018) as measured by cognitive domain of Bayley infant development; socio-emotional development (mean (SD), 75.96 (15.29) vs 67.22 (12.22); mean difference: 8.74; 95% CI 4.91 to 12.57; p-value: <0.0001) as measured by the social-emotional domain of Bayley infant development and on adaptive functioning (mean (SD), 98.05 (17.5) vs 92.76 (13.53); mean difference: 5.29; 95% CI 0.96 to 9.62; p-value: 0.017) as measured by adaptive behaviour domain of Bayley infant development.

Weight in KGs of infants at birth (mean (SD), 2.28 (1.53) vs 1.5 (1.79); mean difference: 0.78; 95% CI 0.3 to 1.25; p-value: 0.002) was higher in the intervention arm, compared with the control arm, according to the birth record of infants collected at 6-month postpartum.

At 12-month postpartum, there were significantly higher scores on home inventory total scores in the intervention arm, compared with the control arm (mean (SD), 38.06 (4.61) vs 33.95 (5.65); mean difference: 4.11; 95% CI 2.69 to 5.52; p-value: <0.0001) as measured by Home Inventory, as well as on its sub-domains including home responsivity (mean (SD), 9.92 (1.41) vs 8.70 (1.86); mean difference: 1.22; 95% CI 0.77 to 1.68; p-value: <0.0001) and home acceptance (mean (SD), 6.77 (1.22) vs 5.46 (1.95); mean difference: 1.31; 95% CI, 0.89 to 1.76; p-value: <0.0001). The findings for breastfeeding and feeding practices at 6 months are presented in online supplemental tables S8.1 and S8.2). No significant differences between groups in the type of first feed, timing of initiation of breastfeeding or type of semi-solid feed were observed.

The analysis of maternal secondary outcomes showed that at 12 month postpartum, the nurturing care intervention was effective, compared with the control intervention, to improve maternal psychological distress (mean (SD), 2 (3.06) vs 3.34 (3.94); mean difference: −1.34; 95% CI −2.32 to −0.36; p-value: 0.007) as measured by the WHO Self-Reporting Questionnaire (SRQ), maternal quality of life in terms of physical health (mean (SD), 75.64 (15.22) vs 68.95 (13.04); mean difference: 6.80; 95% CI 2.29 to 10.68; p<0.0007) and environmental health (mean (SD), 70.15 (16.22) vs 64.34 (16.27); mean difference: 5.49; 95% CI 1.34 to 9.64; p<0.0098) as measured by WHO Quality of Life Questionnaire (WHOQoL) and maternal functioning (mean (SD), 8.01 (11.96) vs 13.26 (12.74); mean difference: −5.24; 95% CI −8.66 to −1.82; p<0.0029) as measured by WHO Disability Assessment Schedule-12 (WHODAS-12 version) in at-risk mothers.

Maternal financial empowerment was comparable between groups at baseline. At 12 months postpartum, there were no significant differences between the intervention and TAU groups in receipt of household money (86.5% vs 86.8%; OR 0.91, 95% CI 0.36 to 2.35; p=0.85) or autonomy in spending (86.5% vs 87.8%; OR 0.64, 95% CI 0.21 to 1.99; p=0.44) (table 4).

**Table 4:** Comparison of maternal financial empowerment between intervention and control groups

Secondary outcomes	Visits	No. (%) of patients with events)		Generalised mixed model analysis	
		Intervention (n=90)	Treatment as usual (n=91)	Odds ratio (95% CI)	P value
Does husband provide money for households?	Baseline	78/87 (89.7)	82/87 (94.3)		
	12 months	77/89 (86.5)	79/91 (86.8)	0.91 (0.36 to 2.35)	0.8508
If yes, does she spend money by will?	Baseline	76/78 (97.4)	80/82 (97.6)		
	12 months	77/89 (86.5)	79/90 (87.8)	0.64 (0.21 to 1.99)	0.4400

The results of the covariate-adjusted analysis show that an infant’s gender, the mother's education and the severity of the mother's psychosocial distress as measured by the SRQ did not influence the intervention effect on the primary outcome (ie, mother-infant interaction) (table 5).

**Table 5:** Summary statistics and results from linear regression model analysis of primary outcome: covariate adjusted analysis*

		Intervention (n=105)		Treatment-as-Usual (n=101)		Linear regression model analysis	
Measurements	Visit	N	Mean (SD)	N	Mean (SD)	Difference (95% CI)	P value
OMCI Total Score	12 months	105	42.05 (8.65)	101	29.74 (8.19)	12.35 (10.03 to 14.68)	<0.0001
Mother Score	12 months	105	28.80 (5.43)	101	20.16 (5.41)	8.67 (7.18 to 10.16)	<0.0001
Child Score	12 months	105	13.25 (3.84)	101	9.58 (3.50)	3.69 (2.67 to 4.70)	<0.0001

* The following covariates are included in the covariate adjusted analysis: gender of the infant, mother’s education and severity of mothers’ psychosocial distress measured using the SRQ measured at baseline.

The results of the subgroup analysis conducted with the pre-specified covariates (gender of the infant, mother’s education, and severity of mothers’ psychosocial distress measured using the SRQ) showed that the mother’s education did not influence the primary outcome (table 6).

**Table 6:** Summary statistics and results from linear regression model analysis of primary outcome: subgroup analysis of primary outcome (n=250)

			N, mean (SD)				
Variable	Subgroup	Visit	Treatment A (n=105)	Treatment B (n=101)	Mean difference (95% CI)	P value	P value from interaction test
OMCI total score							
Gender	Male	12 months	57,42.35 (8.80)	49,28.29 (7.64)	14.07 (10.86 to 17.27)	0.0001	0.1385
	Female	12 months	48,41.69 (8.54)	52,31.12 (8.53)	10.57 (7.18 to 13.96)	0.0001	.
Education	≤Grade 10	12 months	76,42.26 (8.82)	74,28.59 (8.30)	13.67 (10.90 to 16.43)	0.0001	0.0541
	>Grade 10	12 months	29,41.48 (8.30)	27,32.89 (7.12)	8.59 (4.44 to 12.75)	0.0001	.
SRQ total score	≤11	12 months	61,41.52 (8.62)	61,30.48 (8.34)	11.05 (8.01 to 14.09)	0.0001	0.1969
	>11	12 months	44,42.77 (8.74)	40,28.63 (7.94)	14.15 (10.51 to 17.79)	0.0001	.
Mother Score							
Gender	Male	12 months	57,28.91 (5.64)	49,19.31 (5.13)	9.61 (7.52, to 11.70)	0.0001	0.2103
	Female	12 months	48,28.67 (5.23)	52,20.96 (5.59)	7.71 (5.55 to 9.86)	0.0001	.
Education	≤Grade 10	12 months	76,28.87 (5.63)	74,19.30 (5.40)	9.57 (7.79 to 1.35)	0.0001	0.0399
	>Grade 10	12 months	29,28.62 (4.94)	27,22.52 (4.80)	6.10 (3.49 to 8.71)	0.0001	.
SRQ total score	≤11	12 months	61,28.39 (5.43)	61,20.57 (5.55)	7.82 (5.85 to 9.79)	0.0001	0.1911
	>11	12 months	44,29.36 (5.43)	40,19.53 (5.20)	9.84 (7.52 to 2.15)	0.0001	.
Child Score							
Gender	Male	12 months	57,13.44 (3.87)	49,8.98 (3.37)	4.46 (3.05 to 5.87)	0.0001	0.1220
	Female	12 months	48,13.02 (3.83)	52,10.15 (3.56)	2.87 (1.40 to 4.33)	0.0002	.
Education	≤Grade 10	12 months	76,13.39 (3.81)	74,9.30 (3.67)	4.10 (2.89 to 5.30)	0.0001	0.1650
	>Grade 10	12 months	29,12.86 (3.96)	27,10.37 (2.91)	2.49 (0.62 to 4.37)	0.0101	.
SRQ total score	≤11	12 months	61,13.13 (3.76)	61,9.90 (3.55)	3.23 (1.92 to 4.54)	0.0001	0.3030
	>11	12 months	44,13.41 (3.99)	40,9.10 (3.42)	4.31 (2.69 to 5.93)	0.0001	.

Table 7 compares health service utilisation between the intervention and treatment-as-usual (TAU) arms for both mothers and their children over the period of the study. For mothers, private doctors were the most frequently accessed providers, though consultations were slightly higher in the TAU arm (63 vs 50). Use of public hospitals, LHWs and primary healthcare facilities (BHU/RHC) was broadly comparable across arms. Visits to faith healers or homoeopaths were reported in both groups (13 vs 16), while hospital admissions were more frequent in the intervention arm (20 vs 9), although the mean number of admissions was similar (1.15 vs 1.11). Medicine prescription was equal in both groups (71 vs 75), as was use of medical tests (39 vs 33).

**Table 7:** Health service utilisation across two arms at 12 postpartum (mother)

Health service providers accessed	Intervention (*f=xx**)***	TAU (*f=xx**)***
Mother		
LHW	9	10
PHC (BHU/RHC)	24	18
LHV	2	–
CMW	2	3
Public hospital visit	26	20
Private doctor	50	63
Faith healer, homoeopath	13	16
Other health services	11	4
Hospital admissions	20	9
Mean of admissions	1.15	1.11
Medical tests	39	33
Medicine received	71	75
Child		
LHW	7	5
PHC (BHU/RHC)	23	23
Vaccinator (seen privately)	4	4
Public hospital visit	20	29
Private doctor	86	78
Faith healer, homeopath	21	12
Other health services	8	2
Hospital admissions	9	14
Mean of admissions	1.56	1.29
Medical Tests	12	21
Medicine received	89	88
Average duration of consultation (in minutes) M (SD); min-max	51.92 (±38.39); 5–210	46.6 (±41.26); 2–250
Average consultation fee paid (in PKR) M (SD); min-max	650.91 (± 424.91): 0–2500	621.552 (±426.183); 0–3000

.BHU, basic health unit; LHW, lady health visitor; M, mean; RHC, Rural Health Center; SD, standard deviation.

For infants, private doctors were the main source of care, with a higher frequency in the intervention arm (86 vs 78). Public hospital visits were more common in the TAU arm (29 vs 20), whereas faith healer/homeopath visits were higher in the intervention arm (21 vs 12). Hospital admissions were more frequent in the TAU arm (14 vs 9), though the mean number of admissions was slightly greater in the intervention arm (1.56 vs 1.29). Medicine prescription was almost equal across both groups (89 vs 88).

In terms of consultation characteristics, the average consultation duration was longer in the intervention arm (M=51.9 min, SD=38.4) compared with TAU (M=46.6 min, SD=41.3). Average consultation fees were similar across arms (PKR 650.9 vs 621.6).

A total of 1097 individual sessions of intervention were delivered over the period of 18 months, of which 195 sessions (17%) were delivered via telephone due to COVID-19 lockdown in the study sub-district. Out of 125 participants in the intervention arm, 105 (84%) participants received the complete dose of the intervention (15 sessions). The average number of intervention sessions received by each participant was 13 (±1.6). The average length of an in-person session was 40 min (±6.4 min), while the average length of a phone session was 30 min (±5.9 min). Over the period of intervention delivery, a total of 60 weekly supervision meetings (four supervision meetings per month) were conducted by the programme trainers with non-specialist facilitators. The duration of each supervision meeting ranged from 90 to 120 min. During the COVID-19 pandemic, when trainers were conducting sessions via phone, supervision meetings were conducted online. The fidelity of the intervention delivery was assessed by programme trainers using the Enhancing Assessment of Common Therapeutic Factors (ENACT) questionnaire. This included rating 20% of the directly observed in-person sessions (180/902) and 20% of the audio-recorded telephonic sessions (39/195) delivered by each non-specialist. All non-specialists scored a mean score of 3.0 on all domains of the adapted ENACT and an overall percentage of 95% or above on the fidelity checklist. Masking fidelity was assessed by asking assessors to guess allocation status after outcome assessment; correct identification was approximately 10%, suggesting that blinding was successfully maintained.

## Discussion

This study evaluated the effectiveness of a common-elements-based nurturing care intervention in improving mother–infant responsive interaction at 12-month postpartum in rural Pakistan. The findings of the trial showed that compared with the treatment as usual, the intervention significantly enhanced responsive interactions between mothers and infants, with higher OMCI total scores. These findings provide robust evidence that a structured, non-specialist-delivered nurturing care programme can improve caregiver–child relationships during a critical developmental window. Beyond responsive caregiving, the intervention also produced gains in infant developmental outcomes, including cognitive, socio-emotional, and adaptive functioning, highlighting the broader developmental benefits of strengthening maternal capacities in early caregiving. Improvements in maternal psychological well-being, functioning, and quality of life further highlight the intervention’s potential to support the well-being of both mothers and their infants. These findings align with the global nurturing care framework proposed by the Lancet Series on early childhood development, which emphasised the integration of responsive caregiving and maternal support as a pathway to optimise child development.^43^ These findings are also consistent with a recent global scoping review of 138 studies, which identified caregiver mental health as one of the key determinants of responsive caregiving, while highlighting major evidence gaps from low- and middle-income settings.^13^ Our findings extend this evidence by providing trial-based data from an LMIC in South Asia demonstrating that interventions addressing caregivers’ well-being along with improving responsive caregiving with play-based stimulation yield improvements in child development and parenting outcomes.

The responsive stimulation model used in our intervention coached caregivers to engage with their child through play and communication resulting in improved responsive caregiving practices at 12-months postpartum. Responsive caregiving, in which parents provide contingent and supportive responses to a child’s cues, establishes a secure developmental foundation for cognitive, language, and socio-emotional growth.^44^ Play is a particularly powerful avenue for fostering responsiveness, as it encourages parent–child engagement away from daily stressors and creates opportunities for interaction and communication. Evidence from both high- and low-income settings shows that such practices not only enhance child developmental outcomes^44 45^ but also improve caregiver well-being, underlining their critical role in early interventions. An earlier study in Pakistan reported that the responsive caregiving components have the strongest influence on child cognitive outcomes, parenting practices, and the quality of parent–child interactions.^41^ Nurturing care interventions therefore need to move beyond simply providing play materials or offering didactic teaching, and instead emphasise building maternal sensitivity, responsiveness, and agency within the home. The improvements in maternal responsiveness and reductions in maternal psychological distress observed in this study are likely important pathways through which gains in child development are achieved. This is consistent with a recent scoping review, which reported that maternal mental health conditions, including depression and anxiety, were consistently associated with lower sensitivity and responsiveness across diverse settings.^13^ Our approach also aligns with global expert consensus that responsive interaction skills and relationship-building capacities are core competencies for the ECD workforce in LMIC settings.^12 46–48^ By incorporating practice elements that have been consistently identified across effective LMIC early childhood interventions,^23^ our findings contribute to the growing effort to identify scalable, evidence-based components for nurturing care delivery in resource-constrained settings.

The late stages of gestation, particularly the third trimester, present a critical developmental window during which maternal physiological and psychological distress exerts strong influence on fetal growth.^49^ Evidence shows that interventions designed to reduce maternal stress during pregnancy, such as counselling to lessen household workload, promote rest and enhance emotional support, can reduce the incidence of low birth weight and improve mean birth weight in newborns.^50^ Systematic reviews of psychosocial interventions similarly report modest but significant increases in birth weight in intervention groups compared with controls, indicating that alleviating maternal distress can translate into measurable improvements in fetal growth.^51^ In the present trial, we observed higher birth weight in the intervention arm. Although birth weight is influenced by multiple maternal, obstetric, nutritional, socioeconomic and other factors that were not comprehensively assessed or adjusted for, the intervention included components targeting maternal psychological health during pregnancy, which may have contributed to the observed difference. However, this association cannot be attributed definitively to the intervention and should be interpreted cautiously and warrants further investigation.

Our findings align with recent systematic reviews showing that parenting and nurturing care interventions can significantly improve early childhood development outcomes when delivered with adequate intensity and quality. In the present study, the intervention was delivered through structured individual sessions; however, during the COVID-19 pandemic, a proportion of these sessions were delivered telephonically to maintain continuity of the programme while adhering to public health restrictions. A meta-analysis of randomised trials in low- and middle-income countries (LMICs) reported that interventions with structured sessions^52^ and those delivered to vulnerable groups, such as families in rural settings,^49^ were particularly effective in enhancing children’s cognitive development. Consistent with these conclusions, our study demonstrates that a relatively intensive, structured programme focused on responsive caregiving produced measurable benefits for both child development and maternal well-being in low resource settings.

Our findings reinforce the importance of structured workforce preparation when nurturing care interventions are delivered by non-specialists. Embedding trained, supervised and competent non-specialists into routine mother-and-child care pathways enabled the delivery of a nurturing care intervention that simultaneously targeted maternal psychological distress and early responsive interaction, two domains traditionally addressed in separate service streams. This competency-based task-sharing model strengthens sustainability by creating a trained workforce that can be integrated into health systems.

Improvements in maternal well-being, responsive caregiving and infant developmental outcomes, including birth weight, are particularly significant within Pakistani context, where low birth weight remains one of the largest contributors to under-five morbidity and mortality. Delivering core components during the last trimester, a period of heightened vulnerability for fetal growth, highlights the potential value of integrating early psychosocial interventions within MNCH programmes to support maternal and child health. Although a difference in birth weight was observed between the intervention and treatment-as-usual groups, birth weight was a secondary outcome and this finding should be interpreted cautiously given the multiple maternal, obstetric, nutritional, socioeconomic and other factors that influence birth weight and were not comprehensively assessed in this study.

### Strengths and limitations

This study has several notable strengths. First, we developed an integrated nurturing care intervention that simultaneously targeted maternal psychological distress and early responsive interaction, two domains traditionally addressed in separate service streams. Then, we embedded trained, supervised and competent non-specialists into routine mother-and-child care pathways enabling the delivery of the integrated nurturing care intervention in low-resource settings. Importantly, we intervened early, ie, delivered the nurturing care intervention package to at-risk mothers from third trimester until 12-month postpartum, and evaluated its impact in a powered randomised controlled trial design.

Key limitations are (a) endpoint assessments were delayed due to COVID-19 lockdown restrictions, resulting in most infants being assessed at 14 months instead of 12 months, which may have influenced developmental outcome measurements; (b) while we have observed statistically significant effects on a range of secondary outcomes (infant development and maternal outcomes), the study was powered to detect the impact of the nurturing care intervention on primary outcome, ie, responsive caregiving outcome.

## Conclusion

This trial provides robust evidence that an early, integrated, common elements–based nurturing care intervention can simultaneously improve maternal responsive caregiving, maternal psychological well-being and infant developmental outcomes when delivered by trained non-specialists in rural Pakistan. These findings advance the case for embedding maternal mental health and responsive caregiving interventions within existing MNCH platforms, moving beyond vertical programming toward more holistic, community-based models of care.

## Supplementary material

10.1136/bmjopen-2026-119908 online supplemental file 1

10.1136/bmjopen-2026-119908 online supplemental file 2

## Data Availability

Data are available upon reasonable request.
