# Supplementary material for: Common elements-based nurturing care intervention to improve mother–infant responsive interaction and maternal psychological wellbeing in rural Pakistan: findings of a single-blinded individual randomised controlled trial
Source: BMJ Open. 2026 Oct 8;16(10):e119908. doi: 10.1136/bmjopen-2026-119908 (PMC13656113; doi:10.1136/bmjopen-2026-119908)
Supplement: Supplementary file 2 [file bmjopen-2026-119908.reviewer_comments.pdf]

PEER REVIEW HISTORY

BMJ Open publishes all reviews undertaken for accepted manuscripts. Reviewers are asked to complete a checklist review form and are provided with free text boxes to elaborate on their assessment. These free text comments are reproduced below.

ARTICLE DETAILS

Title (Provisional)

Common Elements-Based Nurturing Care Intervention to Improve Mother-Infant Responsive Interaction and Maternal Psychological Wellbeing in Rural Pakistan: Findings of a Single-blinded Individual Randomized Controlled Trial (RCT)

Authors

Hamdani, Syed Usman; Huma, Zill-e; Gillani, Ayella; Jamil, Huda; Zafar, Syeda Wajeeha; Kahloon, Lubna Ejaz; Shafique, Fakhira; Javed, Hashim; Wang, Duolao; Rahman, Atif

---

VERSION 1 - REVIEW

**Reviewer**	**1**
**Name**	**Glover, Vivette**
**Affiliation**	**Imperial College London, IRDB**
**Date**	**03-Jun-2026**
**COI**	**None**

This is a very good account of an interesting and potentially important trial.

Two points.

Were the assessors of the outcomes (especially CCI) blind to treatment or control. This should be stated.

Did the intervention include any advice about nutrition? if so what? Did the authors find the size of the effect on birthweight surprising? This could be discussed further.

**Reviewer**	**2**
**Name**	**Anderson, James R**
**Affiliation**	**University of Nebraska Medical Center, Biostatistics**
**Date**	**03-Jun-2026**
**COI**	**None**

Lines 298 and following: The authors describe each of the assessment tools used, but a reference for each would be helpful (e.g., https://iris.who.int/server/api/core/bitstreams/47036142-21c6-403d-be69-2704380ae86a/content for SRQ-20).

Lines 398-401: The authors need to describe the method(s) used for imputation (if used). The Table 2 (page 22) sample sizes suggest that the primary and secondary endpoints were calculated using the known data without imputation.

Table 2 (page 22): I assume that the "effect size" reported are Cohen's d (e.g., [mean difference / est(standard deviation of the data]). This should be made explicit somewhere in the Table.

Table 2 lines 12-14: Why are the effect size confidence intervals for OMCI backwards (bigger value first)?

Page 23, lines 11-12: Ages and Stages: What does the (12-months, N=8) associated with the label mean?

Page 23, line 13 and following into page 24: It appears there is much more 12-month data for some of the Ages and Stages than others. All of the comparisons are of small subsets of the randomized subjects and many have sample sizes (e.g., 6 and 2; 5 and 3) that make the comparisons meaningless. I suggest that all of the comparisons with maximum sample size (both groups) less than (say) 80 be eliminated and indicate that in the text.

VERSION 1 - AUTHOR RESPONSE

Reviewer: 1
Dr. Vivette Glover, Imperial College London
Comments to the Author:
This is a very good account of an interesting and potentially important trial.
Two points.
Comment 8: *Were the assessors of the outcomes (especially CCI) blind to treatment or control. This should be stated.*

Response: Thank you for highlighting this. The manuscript already stated that the assessment team conducting the baseline and follow-up assessments was blinded to treatment allocation (page 6, lines 229–230).

Comment 9: *Did the intervention include any advice about nutrition? if so what? Did the authors find the size of the effect on birthweight surprising? This could be discussed further.*

Response: Thank you for this important comment. Yes, the intervention included nutrition and feeding-related advice as part of the Nurturing Care package. This included guidance on the introduction of solid foods, the concept of "super foods," food and hygiene, continuation of breastfeeding and weaning at 6 months, as well as vaccination. We have clarified these components in the revised manuscript (please see page 6, lines 256-258). We have also

expanded the Discussion to acknowledge the observed difference in birth weight and to interpret this finding cautiously, given that birth weight was a secondary outcome and can be influenced by multiple factors.

Reviewer: 2
Dr. James R Anderson, University of Nebraska Medical Center
Comments to the Author:
Comment 10: *Lines 298 and following: The authors describe each of the assessment tools used, but a reference for each would be helpful (e.g., https://iris.who.int/server/api/core/bitstreams/47036142-21c6-403d-be69-2704380ae86a/content for SRQ-20).*

Response: Thank you for this helpful suggestion.

We have added appropriate references for each of the assessment tools used in the study, including the SRQ-20, to support their use and scoring.

Comment 11: *Lines 398-401: The authors need to describe the method(s) used for imputation (if used). The Table 2 (page 22) sample sizes suggest that the primary and secondary endpoints were calculated using the known data without imputation.*

Response: Thank you for raising this point. We discussed this with the trial statistician and clarified the imputation approach. The relevant text has been revised in the manuscript.

Comment 12: *Table 2 (page 22): I assume that the "effect size" reported are Cohen's d (e.g., [mean difference / est(standard deviation of the data]). This should be made explicit somewhere in the Table.*

Response:

Thank you for highlighting this.

We have clarified in the revised manuscript and Table 2 that the reported effect sizes are Cohen's d. The corresponding table footnote has been updated accordingly.

"Effect size was calculated as Cohen's d, based on the adjusted mean difference divided by the estimated pooled standard deviation."

Comment 13: *Table 2 lines 12-14: Why are the effect size confidence intervals for OMCI backwards (bigger value first)?*

Response: Thank you for noting this. We discussed this with the trial statistician and have update the Table 2.

Comment 14: *Page 23, lines 11-12: Ages and Stages: What does the (12-months, N=8) associated with the label mean?*

Response: Thank you for highlighting this. The notation "12-months, n=8" referred to the total number of children with ASQ-3 data available in the 12-month age category.

Comment 15: *Page 23, line 13 and following into page 24: It appears there is much more 12-month data for some of the Ages and Stages than others. All of the comparisons are of small subsets of the randomized subjects and many have sample sizes (e.g., 6 and 2; 5 and 3) that make the comparisons meaningless. I suggest that all of the comparisons with maximum sample size (both groups) less than (say) 80 be eliminated and indicate that in the text.*

Response: Thank you for this important observation. We agree that the ASQ-3 comparisons based on very small age-specific subsamples are not sufficiently informative. We have therefore removed comparisons where the maximum total sample size across both treatment groups was less than 80 and clarified this in the Results section.

Please see table 2.

VERSION 2 - REVIEW

**Reviewer**	**2**
**Name**	**Anderson, James R**
**Affiliation**	**University of Nebraska Medical Center, Biostatistics**
**Date**	**01-Sep-2026**
**COI**	

All of the issues I raised in my initial review have been adequately addressed.
