# Supplementary material for: Common elements-based nurturing care intervention to improve mother–infant responsive interaction and maternal psychological wellbeing in rural Pakistan: findings of a single-blinded individual randomised controlled trial
Source: BMJ Open. 2026 Oct 8;16(10):e119908. doi: 10.1136/bmjopen-2026-119908 (PMC13656113; doi:10.1136/bmjopen-2026-119908)
Supplement: Supplementary file 3 [file bmjopen-16-10-s002.docx]

**Supplementary Material**

**Table S8.1: Feeding practices at 6 months postpartum**

| **Feeding practice** | **Category** | **Intervention, n (%)** | **TAU, n (%)** | **p value** |
| --- | --- | --- | --- | --- |
| **Child's first feed after birth** |  |  |  | **0.7525** |
|  | Ghutti *(traditional herbal preparation given to newborns)* | 38 (40.0) | 32 (33.7) |  |
|  | Mother feed *(breast milk)* | 40 (42.1) | 41 (43.2) |  |
|  | Powdered milk *(infant formula)* | 16 (16.8) | 21 (22.1) |  |
|  | Water *(water)* | 1 (1.1) | 1 (1.1) |  |
| **When was mother feeding first initiated?** |  |  |  | **0.4497** |
|  | After 24 hours | 18 (18.9) | 21 (22.1) |  |
|  | Mother feeding not started *(breastfeeding not initiated)* | 5 (5.3) | 10 (10.5) |  |
|  | Within 1 hour | 18 (18.9) | 13 (13.7) |  |
|  | Within 1–5 hours | 40 (42.1) | 41 (43.2) |  |
|  | Within 12–24 hours | 9 (9.5) | 4 (4.2) |  |
|  | Within 6–11 hours | 5 (5.3) | 6 (6.3) |  |
| **Type of semi-solid feed** |  |  |  | **0.5107** |
|  | Halwa *(sweet semolina-based food)* | 1 (1.3) | 0 (0) |  |
|  | Cerelac *(commercial infant cereal)* | 41 (54.7) | 45 (65.2) |  |
|  | Custard *(custard)* | 2 (2.7) | 3 (4.3) |  |
|  | Daliya *(porridge)* | 15 (20.0) | 8 (11.6) |  |
|  | Mashed banana *(mashed banana)* | 10 (13.3) | 10 (14.5) |  |
|  | Other *(other foods)* | 6 (8.0) | 3 (4.3) |  |
| **Type of solid feed** |  |  |  | **0.0002** |
|  | Egg *(egg)* | 1 (1.6) | 0 (0) |  |
|  | Khichri *(rice and lentil porridge)* | 4 (6.6) | 1 (3.2) |  |
|  | Other *(other solid foods)* | 0 (0) | 10 (32.3) |  |
|  | Rice | 10 (16.4) | 4 (12.9) |  |
|  | Roti *(flatbread)* | 46 (75.4) | 16 (51.6) |  |

Note: Values are n (%). p values are from χ² tests. Analysis was based on the intention-to-treat population. The original table reports the feeding-practice categories and corresponding ITT results.

**Table S8.2: Feeding practices at 6 months postpartum — Per-protocol analysis**

| **Feeding practice** | **Category** | **Intervention, n (%)** | **TAU, n (%)** | **p value** |
| --- | --- | --- | --- | --- |
| **Child's first feed after birth** |  |  |  | **0.9635** |
|  | Ghutti *(traditional herbal preparation given to newborns)* | 32 (37.6) | 31 (36.0) |  |
|  | Mother feed *(breast milk)* | 36 (42.4) | 35 (40.7) |  |
|  | Powdered milk *(infant formula)* | 16 (18.8) | 19 (22.1) |  |
|  | Water *(water)* | 1 (1.2) | 1 (1.2) |  |
| **When was mother feeding first initiated?** |  |  |  | **0.2282** |
|  | After 24 hours | 13 (15.3) | 19 (22.1) |  |
|  | Mother feeding not started *(breastfeeding not initiated)* | 4 (4.7) | 10 (11.6) |  |
|  | Within 1 hour | 17 (20.0) | 11 (12.8) |  |
|  | Within 1–5 hours | 37 (43.5) | 37 (43.0) |  |
|  | Within 12–24 hours | 9 (10.6) | 4 (4.7) |  |
|  | Within 6–11 hours | 5 (5.9) | 5 (5.8) |  |
| **Type of semi-solid feed** |  |  |  | **0.4338** |
|  | Halwa *(sweet semolina-based food)* | 1 (1.5) | 0 (0) |  |
|  | Cerelac *(commercial infant cereal)* | 37 (56.1) | 40 (64.5) |  |
|  | Custard *(custard)* | 1 (1.5) | 3 (4.8) |  |
|  | Daliya *(porridge)* | 13 (19.7) | 7 (11.3) |  |
|  | Mashed banana *(mashed banana)* | 8 (12.1) | 9 (14.5) |  |
|  | Other *(other foods)* | 6 (9.1) | 3 (4.8) |  |
| **Type of solid feed** |  |  |  | **0.0002** |
|  | Egg *(egg)* | 1 (1.9) | 0 (0) |  |
|  | Khichri *(rice and lentil porridge)* | 3 (5.7) | 1 (3.6) |  |
|  | Other *(other solid foods)* | 0 (0) | 10 (35.7) |  |
|  | Rice | 7 (13.2) | 2 (7.1) |  |
|  | Roti *(flatbread)* | 42 (79.2) | 15 (53.6) |  |
| **Delivery type** |  |  |  | **0.6120** |
|  | Normal *(vaginal delivery)* | 34 (40.0) | 31 (36.0) |  |
|  | Episiotomy *(vaginal delivery with episiotomy)* | 20 (23.5) | 26 (30.2) |  |
|  | Operation *(caesarean section)* | 31 (36.5) | 29 (33.7) |  |
